# Redox Active α-Lipoic Acid Differentially Improves Mitochondrial Dysfunction in a Cellular Model of Alzheimer and Its Control Cells

**DOI:** 10.3390/ijms23169186

**Published:** 2022-08-16

**Authors:** Fabian Dieter, Carsten Esselun, Gunter P. Eckert

**Affiliations:** Biomedical Research Center, Institute of Nutritional Sciences, Justus-Liebig-University of Giessen, Schubert-Street 81, D-35392 Giessen, Germany

**Keywords:** alpha lipoic acid, Alzheimer disease, mitochondria, ROS, mitochondria dysfunction, respiratory chain

## Abstract

Introduction: Alpha lipoic acid (ALA) is a sulphur-containing organic compound, derived from octanoic acid, and an important cofactor for mitochondrial respiratory enzymes. It has strong antioxidant properties that improve mitochondrial function. We investigated if ALA improves mitochondrial dysfunction in a cellular model of Alzheimer’s disease (AD). Methods: SH-SY5Y-APP_695_ cells were used as a model for an early stage of AD. Vector-transfected SH-SY5Y-MOCK cells served as controls. Using these cells, we investigated mitochondrial respiration (OXPHOS), mitochondrial membrane potential (MMP), adenosine triphosphate (ATP) production, and citrate synthase activity (CS) in cells treated with ALA. Cells were treated for 24 h with different concentrations of ALA and with or without the complex I inhibitor rotenone. Results: Incubation with ALA showed a significant increase in ATP levels in both SH-SY5Y-APP_695_ and SH-SY5Y-MOCK cells. MMP levels were elevated in SH-SY5Y-MOCK cells, treatment with rotenone showed a reduction in MMP, which could be partly alleviated after incubation with ALA in SH-SY5Y-MOCK cells. ALA treatment showed significant differences in respiration chain complex activities in SH-SY5Y-MOCK cells. Citrate synthase activity was unaffected. ROS levels were significantly lower in both cell lines treated with ALA. Conclusions: ALA increased the activity of the different complexes of the respiratory chain, and consequently enhanced the MMP, leading to increased ATP levels indicating improved mitochondrial function. ALA only marginally protects from additional rotenone-induced mitochondrial stress.

## 1. Introduction

**Scheme 1:**
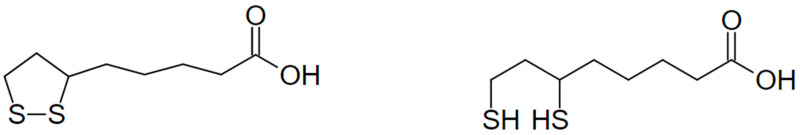
Lipoic acid and dihydrolipoic acid structure: lipoic acid (1,2-dithiolane-3-pentanoicacid) is an eight-carbon dithiol compound with a high reduction potential. Its reduced form is dihydrolipoic acid.

Alzheimer’s disease (AD) is a slowly progressing neurodegenerative disorder that starts with mild memory loss and, as it progresses, culminates in severe impairment of executive and cognitive functions [1,2]. The mean prevalence of AD exponentially increases with age. It is 1.6% for those aged 65–69, 15.6% for those aged 80–84, and 44.17% for those aged 90. More than 50 million patients worldwide suffer from AD, and projections for the year 2050 show that there will be more than 152 million AD patients [3]. The likelihood of developing dementia increases with each additional year of life [4]. Despite extensive research into the pathology, there is currently no cure for AD. AD is classically characterized by two hallmark pathologies: extracellular β-amyloid plaque deposition and intracellular deposition of neurofibrillary tangles of hyperphosphorylated tau [5]. Mitochondrial dysfunction (MD) is increasingly recognized as one of the early events in the progression of Alzheimer’s disease. Disturbed bioenergetics leading to MD are likely to play an important role in aging and subsequently in AD [6,7]. For example, oxidative phosphorylation, the mitochondrial membrane potential (MMP), and the ATP supply of the cells are influenced by AD [8,9,10]. It, therefore, seems reasonable to pursue both preventive and therapeutic approaches. A schematic representation of ALA is shown in Scheme 1.

A promising molecule is alpha lipoic acid (ALA). ALA is widely used as a pharmaceutical and nutraceutical due to its strong antioxidant and anti-inflammatory properties [11,12,13].

ALA is synthesised in mitochondria but is also ingested as food or food supplements. Na^+^/multivitamin transporter (hSMVT) is responsible for the transport of ALA. However, in vitro evidence suggests the existence of more than one cellular transport mechanism involving other fatty acid transporters, such as the monocarboxylic acid transporter (MCT) [14]. ALA contains two thiol groups, which are the main reason for its chemical activity. Due to its amphiphilic character and the ability to cross the blood-brain barrier, protection is afforded to both intracellular and extracellular environments [15].

These molecular properties of ALA give it the ability to positively influence a variety of diseases and redox imbalances. An imbalance in transition metals can lead to elevated reactive oxygen species (ROS). ALA and its reduced form, dihydrolipoic acid (DHLA), can chelate these metals. In addition, heavy metals are also detoxified by ALA and DHLA [16,17]. There are also benefits to diabetic neuropathy and impaired glucose uptake, and the regeneration of glutathione (GSH) and vitamins C and E [18,19,20,21,22].

ALA seems to have a positive effect on neurodegenerative diseases such as AD. ALA improves cognitive performance and could be considered as a promising bioactive substance for AD by affecting multiple mechanisms such as: (1) impaired acetylcholine production; (2) hydroxyl radical formation, ROS production, and neuroinflammation; (3) impaired amyloid plaque formation; (4) decreased glutathione expression; and (5) impaired neurotransmitter levels [23]. Several cell and animal studies [24,25,26] have demonstrated beneficial effects and even some smaller human intervention studies showed promising results [27,28,29].

Factors such as amyloid beta (Aβ)-induced mitochondrial dysfunction can lead to a ROS formation and a deterioration of mitochondrial energy metabolism. ALA is a molecule that combines many positive properties. These properties offer the possibility of having a positive impact on mitochondria and thus on neurodegenerative diseases. We investigated the effects of ALA on oxidative phosphorylation, MMP, ATP levels, and citrate synthase activity in SH-SY5Y-MOCK and SH-SY5Y-APP_695_ cells.

## 2. Results

### 2.1. Effect of Alpha Lipoic Acid on Adenosine Triphosphate Levels

**Figure 1:**
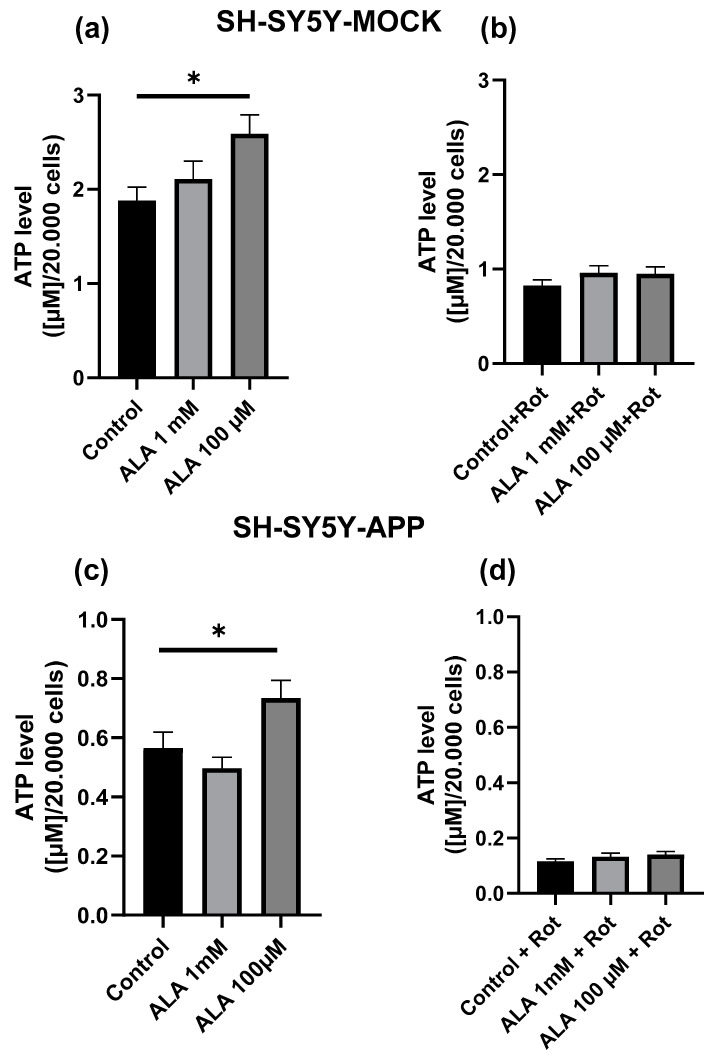
ATP levels in SH-SY5Y-MOCK after alpha lipoic acid (ALA) treatment. (**a**) Cells were incubated with ALA at different concentrations for 24 h and measured against cells treated with ethanol the solvent of ALA. (**b**) ATP levels in rotenone (Rot) [25 µM]-treated SH-SY5Y-MOCK cells. Before rotenone impairment, SH-SY5Y-MOCK cells were incubated with either ALA 100 µM, 1 mM, or ethanol for 1 h before exposure to 25 μM rotenone for another 24 h. SH-SY5Y-APP_695_ were treated likewise. (**c**) Treatment with ALA. (**d**) Additional rotenone. Data are displayed as the means ± SEM. N = 17–22. Statistical significance was tested via one-way ANOVA and Tukey’s post-hoc test (* *p* < 0.05).

In neither SH-SY5Y-APP_695_ nor in SH-SY5Y-MOCK cells, incubation with 1 mM ALA for 24 h showed a significant effect on basal ATP level. Incubation with 100 µM ALA showed a significant increase in both SH-SY5Y-APP_695_ (*p* < 0.0417) and SH-SY5Y-MOCK (*p* < 0.0234) cells. To induce additional mitochondrial stress, complex I was inhibited using rotenone, which increases ROS production common in AD [30]. None of the ALA concentrations used (100 µM; 1 mM) showed a protective effect against complex I impairment in the cell lines used, although 100 µM ALA had a beneficial effect on the basal ATP level as displayed in Figure 1.

### 2.2. Mitochondrial Membrane Potential

**Figure 2:**
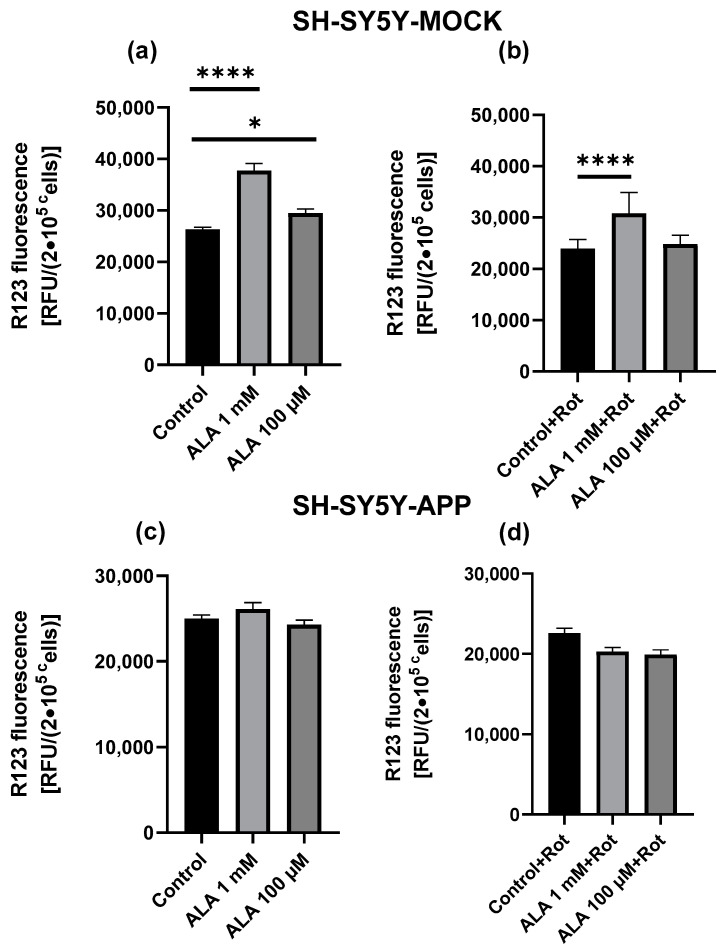
Effects of alpha lipoic acid (ALA) on mitochondrial membrane potential (MMP) in SH-SY5Y-MOCK. (**a**) Cells were incubated with ALA at different concentrations for 24 h and measured against cells treated with ethanol the solvent of ALA. (**b**) MMP in rotenone (R) [25 µM] treated SH-SY5Y-MOCK cells. Before rotenone impairment, SH-SY5Y-MOCK cells were incubated with either ALA 100 µM, 1 mM, or ethanol for 1 h before exposure to 25 μM rotenone for another 24 h. SH-SY5Y-APP_695_ cells were treated in the same way. (**c**) Treatment with ALA and ethanol. (**d**) Additional damage with rotenone. Data are displayed as the means ± SEM. N = 12–23. Statistical significance was tested via one-way ANOVA and Tukey’s post-hoc test (**** *p* < 0.0001, * *p* < 0.05).

The basal MMP levels of SH-SY5Y-MOCK cells were significantly higher if treated with ALA 1 mM (*p* < 0.0001) or ALA 100 µM (*p* = 0.0127) (Figure 2a). Treatment with rotenone showed a reduction in MMP, which could be partly alleviated by ALA 1 mM (*p* < 0.0001) (Figure 2c). However, ALA 100 µM, on the other hand, had no effects. MMP levels of SH-SY5Y-APP_695_ cells were unaffected by ALA treatment (Figure 2b,d).

### 2.3. Respiration

**Figure 3:**
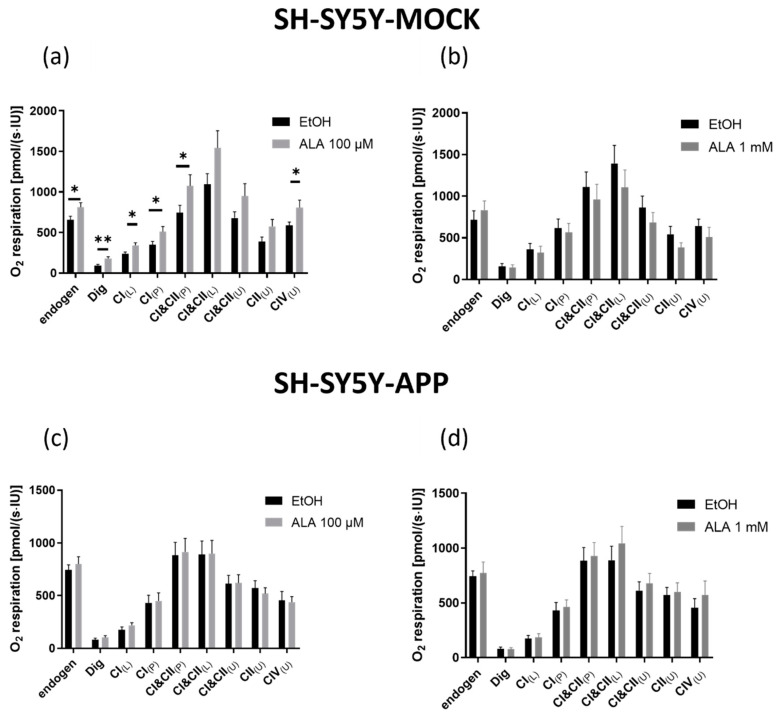
Mitochondrial respiration in SH-SY5Y-MOCK after incubation with alpha lipoic acid (ALA) or solvent control ethanol (EtOH). A solution containing 10^6^ cells/mL was used to measure the oxygen consumption of cells in an Oxygraphy-2k. SH-SY5Y-MOCK cells were incubated with ALA 100 µM (**a**) or ALA 1 mM (**b**) and compared with a control group treated with EtOH. Respectively, SH-SY5Y-APP_695_ cells were incubated with ALA 100 µM (**c**) or ALA 1 mM (**d**) and compared with a control group treated with EtOH. To investigate the activity of the complexes in the respiratory chain, several inhibitors, substrates, and uncouplers were added. The addition of a substance was done as follows. Dig = addition of digitonin; CI_(L)_ leak respiration of complex I; CI_(P)_ coupled respiration of complex I; CI&CII_(P)_ physiological respiration; CI&CII_(L)_ leak respiration of complexes I and II; CI&CII_(U)_uncoupled respiration displaying maximum activity of complexes I and II. CII_(U)_ uncoupled respiration of complex II; CIV_(U)_ uncoupled respiration. Data are displayed as the means ± SEM. N = 12–15. Statistical significance was tested via student’s *t*-test (** *p* < 0.01 and * *p* < 0.05).

Incubation of SH-SY5Y-MOCK cells with ALA 1 mM for 24 h did not affect the respiration of the complexes (Figure 3b). The treatment with 100 µM ALA, however, showed significant differences in complex activity. Endogenous respiration (*p* < 0.034), respiration after permeabilization (*p* < 0.002), leak respiration of complex I (*p* < 0.0128), coupled respiration of complex I (*p* < 0.0436), coupled respiration of complexes I&II (*p* < 0.0454), and uncoupled respiration of complex IV (*p* < 0.0347) showed an increase in complex activity compared with the control (Figure 3a). SH-SY5Y-APP_695_ cells were unaffected by ALA incubation (Figure 3c,d).

### 2.4. Citrate Synthase Activity

**Figure 4:**
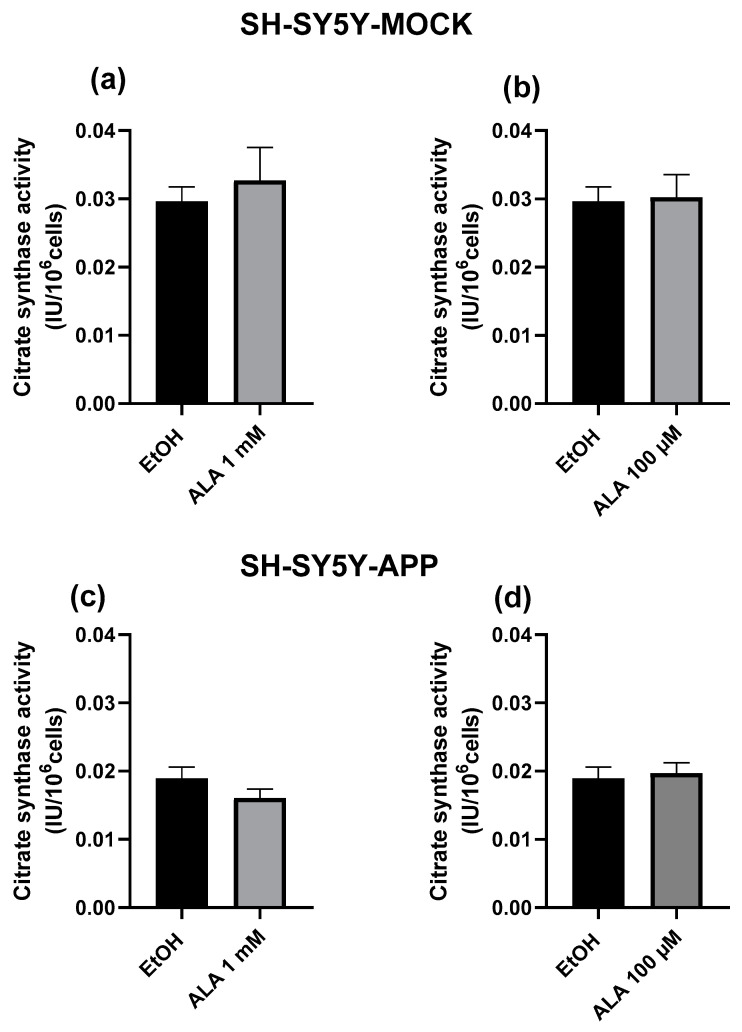
Citrate synthase activity of SH-SY5Y-MOCK cells after 24 h incubation with (**a**) 100 µM alpha lipoic acid (ALA) or (**b**) 1 mM ALA, ethanol served as a control in both cases. Citrate synthase activity of SH-SY5Y-APP_695_ cells after 24 h incubation with (**c**) 100 µM alpha lipoic acid (ALA) or (**d**) 1 mM ALA, ethanol served as control. Data are displayed as the means ± SEM. N = 10–15. Statistical significance was tested via student’s *t*-test.

Citrate synthase activity is a known marker for mitochondrial content [31]. ALA treatment showed no significant difference between treatment and control in both cell lines and ALA concentrations, this is shown in Figure 4.

### 2.5. ROS Measurement

**Figure 5:**
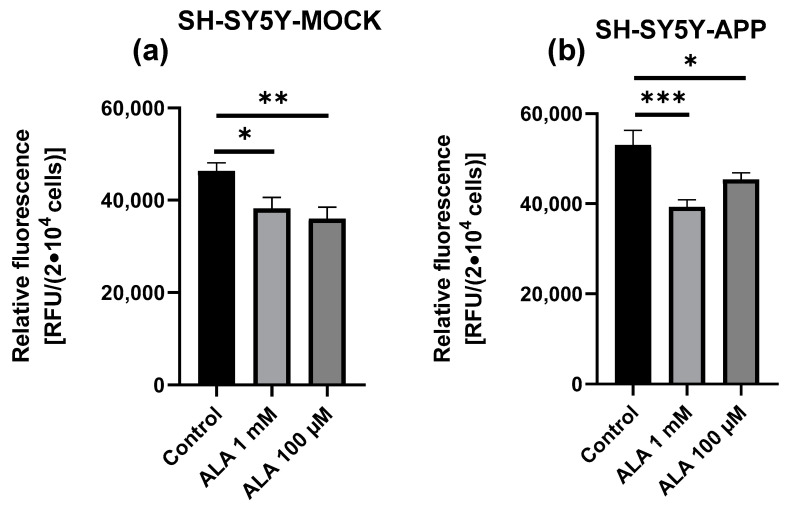
Effect of alpha lipoic acid (ALA) on ROS production in SH-SY5Y-APP and MOCK cells. ROS production of SH-SY5Y-MOCK cells (**a**) after 24 h incubation with 100 µM ALA or 1 mM ALA, ethanol served as a control in both cases. ROS production of SH-SY5Y-APP cells (**b**) after 24 h incubation with 100 µM ALA or 1 mM ALA, ethanol served as a control in both cases. Data are displayed as the means ± SEM. N = 8. Statistical significance was tested via one-way ANOVA and Tukey’s post-hoc test (*** *p* < 0.001, ** *p* < 0.01 and * *p* < 0.05).

ALA treatment showed a significant difference between treatment and control in both cell lines and ALA concentrations in all groups, with ALA 1 mM (*p* < 0.0316) or ALA 100 µM (*p* = 0.0064) in SY5Y-MOCK (Figure 5a), and ALA 1 mM (*p* < 0.0006) or ALA 100 µM (*p* = 0.0437) in SY5Y-APP cells (Figure 5b). In SY5Y-MOCK cells, 100 µM ALA shows a stronger effect, whereas in SY5Y-APP, 1 mM ALA shows a stronger effect. The comparison of both cell lines shows that SY5Y-APP cells have higher levels of ROS.

## 3. Discussion

Advancing age leads to mitochondrial dysfunction and also plays a major role in the pathogenesis of neurodegenerative diseases such as Alzheimer’s disease (AD) [7,32,33]. Dysfunctional mitochondria show elevated ROS levels [34] and impaired function, such as lowered oxidative capacity, reduced oxidative phosphorylation, and decreased ATP production [6,7].

As a model of early Alzheimer’s Disease, we used SH-SY5Y-APP_695_ cells; these cells are transfected with neuronal APP, which leads to increased production of cerebral Aβ, a neurotoxic peptide expressed in AD [35]. SY5Y-APP_695_ showed mitochondrial dysfunction indicated by reduced ATP, MMP, and citrate synthase activity, and generally lower respiration in the complexes of the respiratory chain. In addition to the generally lower respiration of complexes in SY5Y-APP_695_ cells, the buffering capacity of maximal respiration is also greatly reduced. Uncoupling CI and CII by FCCP resulted in only a small increase in respiration in SY5Y-APP_695_ cells compared with respiration in the coupled state. On the other hand, uncoupling in SY5Y-MOCK cells led to an increase of respiration, with the capacity to further improve. These findings are in line with former results from our group and others regrading elevated APP production [6,36,37,38,39,40].

In SH-SY5Y-MOCK, all concentrations of ALA increased ATP levels, with ALA 100 µM doing so significantly. The same significant effect could be observed in SY5Y-APP_695_ cells, whilst 1 mM had no effect. Other studies reported similar increased ATP levels after adding ALA in cells and rats. 100 µM ALA was used in a SH-SY5Y Parkinson model and 100 mg/kg body weight/day ALA was dissolved in alkaline saline (0.5%) and fed to male albino rats of Wistar strain [41,42]. With elevated ATP production via the respiratory chain, there is not only an increase in energy output, but also in ROS production [43,44]. One possible explanation for the effect of ALA on ATP levels could be its antioxidant properties, which can scavenge the excess ROS produced [45]. These scavenging properties might protect cells against ROS-induced damage. Other groups showed, in SH-SY5Y cells and other cell lines, that ALA can prevent cells from oxidative damage due to its antioxidative capacity [46,47]. This protection could be a cause of the increased ATP level compared with untreated controls. On the other hand, ALA could not protect both cell lines from rotenone-induced damage. A tendency towards increased ATP levels could be observed, which, however, was not significant. The well-described antioxidative effect of ALA [48,49] may be the basis of the observed effect on ATP levels and complex activity. To confirm our hypothesis that ALA has a protective effect against ROS, we measured ROS levels in SY5Y-MOCK and SY5Y-APP_695_ cells. We found that ALA reduced ROS in both cell lines at both concentrations. This leads to the conclusion that the reduction of ROS may help with higher complex activity and ATP levels. However, the effects shown are not solely due to the protective effect against ROS. Therefore, there could also be other underlying mechanisms that could explain the positive effects of ALA. Zhang et al. showed that cells incubated with 100 µM ALA for four weeks exhibited increased *SIRT1* and *PGC-1α* expression [50]. Through deacetylation of *PGC1*, *SIRT1* influences energy metabolism and more energy is produced by increasing mitochondrial biogenesis [39,51]. To take the lower incubation time into account, another possible reason could be a stimulated glycolysis via ALA [52].

The mitochondrial membrane potential (MMP) is generated by proton pumps (Complexes I, III, and IV). Together with the proton gradient, MMP forms the transmembrane potential of hydrogen ions which is harnessed to make ATP [53]. Mitochondria are the main producers and due to their spatial proximity, also the main targets of ROS. The ROS produced in the respiratory chain have a negative effect on the functions and the MMP in the mitochondria. These impairments are caused by strand breaks of the DNA, lipid peroxidation, and inactivation of enzymes. ROS are scavenged in the mitochondria by various mechanisms, such as the enzymes superoxide dismutase, catalase, and glutathione peroxidase, but also antioxidant molecules such as vitamin E [54,55,56]. Due to its strong antioxidant properties [57], α-lipoic acid, similar to other antioxidants, can ensure that damage caused by excessive ROS levels is prevented. An improvement of MMP by ALA was also shown by McCarty et al. in hepatic mitochondria isolated from aged rats. These received an ALA-enriched diet (0.5% *w*/*w*) for up to 1 month. Treatment with ALA was able to induce regeneration of the mitochondria of aged rats and raise their MMP to the level of young rats [58]. In addition, there was a reduced ROS formation. Similar results were also observed by Ames and Liu [59]. The higher MMP levels of the ALA-treated SH-SY5Y-MOCK cells measured in this work could be explained by a reduction in ROS and an associated improvement in ETS. This improvement can also be seen in improved respiration and ATP production. The same effects could not be observed in SH-SY5Y-APP_695_ cells. Here the MMP was unaffected by ALA treatment. As mentioned earlier, the general lower respiration of complexes in SY5Y-APP_695_ cells leads to a lower overall OXPHOS and, therefore, lower energy production. Although in previous work we showed that ROS level in APP_695_ cells was higher [36,38], here ALA does not seem to have a positive effect on APP_695_ cells. Inhibition of complex-I by rotenone, leading to increased ROS [60], resulted in a drop of MMP and ATP in both cell lines. In contrast to ATP levels, ALA treatment in SH-SY5Y-MOCK resulted in an increase in MMP. However, this did not lead to an increase in energy production.

Due to AD, but also due to ageing, the functionality of mitochondria and the activity of its respiratory complexes decreases. In AD, complexes I and IV are particularly affected. Due to these functional limitations, there is a reduced production of ATP and, thus, a reduced energy metabolism in the cells [61,62]. Impaired function of the complexes leads to increased ROS production and, finally, to apoptosis of the mitochondria [63]. SH-SY5Y-MOCK cells treated with 100 µM ALA showed an increased activity in all complexes. Other concentrations and the SY5Y-APP_695_ model were not affected. Palaniappan and Dai showed that ALA increased the performance of brain mitochondria in both young and old rats. This improvement in the complex activities was significantly greater in the old rats and occurred in all complexes. In the young rats, improved function was seen only in complexes I, III, and IV [41]. Similar results with an improved effect of complexes I and IV were found in mitochondria of the hearts of old rats [64]. The increased complex activity, compared with the control, seems to have a positive effect on energy production in the SY5Y-MOCK cells. The positive effects on the respiratory chain are also reflected in the ATP values. These increased levels of ATP can result from the increased respiration of the cells with ALA. However, the increased activity of the complexes can also lead to increased oxidative stress. The main producers of ROS in the ETS are complexes I and III; through this ROS production, the complexes and the downstream complex IV can be negatively influenced [65]. This additional stress caused by the increased activity of the complexes would have to be compensated by the mitochondria in order to gain an advantage from the increased activity. Here, the antioxidant properties of ALA could help to intercept the additional ROS that arise and thus ensure that these do not have a negative effect on the function of the complexes. In a study also conducted with SY5Y cells, Song et al. found positive effects of ALA 100 µM on cells damaged with acrylamide (ACR). ACR leads to mitochondrial dysfunction, an imbalance in redox status, and ultimately cell death. ALA was shown to negate the negative effects of ACR through its antioxidant effects and increased expression and regeneration of antioxidant proteins, such as SIRT1 [66]. Moreover, ALA has also been shown to restore the expression of OXPHOS complexes in HepG2 cells, ranging in a concentration between 0.5–2 mM [67]. These results point to further protective properties of ALA, possibly due to the activation of various signalling pathways in cells. On the other hand, there seems to be a limitation. ALA 1 mM elevated MMP and ATP in SY5Y-MOCK but did not affect complexes of the respiratory chain. If higher amounts of ALA are considered, they do not seem to have any further positive effect on the reduction of ROS, suggesting that a plateau occurs. Lee and colleagues were able to show that ALA has different effects on cells depending on the dose. ALA had a toxic effect on the cells used in higher concentrations (300–1200 µM). However, this effect was reversed by additional damage with H_2_O_2_, and ALA now had a protective effect on the cells. Possible positive effects of ALA outside of scavenging damage by ROS could be counteracted by the too-high dose of ALA (1 mM), as this dose may have other negative effects [68]. The same is found for SY5Y-APP_695_ cells, again, there is no effect on oxygen consumption. Others found protective effects from ALA regarding Aβ-induced damage. DHLA in concentrations spanning from 25–200 µM was able to protect dissociated primary hippocampal cultures from damage caused by Aβ 25–35, iron, and hydrogen peroxide [69]. It was described that pre-treatment with ALA led to improved cell survival rates, whilst simultaneous treatment led to an increase in free radical generation and cell death. Ono et al. showed that ALA inhibits the formation from fresh Aβ, as well as destabilizes pre-formed Aβ in vitro [70].

## 4. Materials and Methods

### 4.1. Chemicals

The chemicals used for this research were purchased from either Merck (Kenilworth, NJ, USA), Sigma Aldrich (St. Louis, MO, USA), Thermo Fisher Scientific (Waltham, MA, USA), or VWR (Radnor, PA, USA) in the highest purity available. (±)-α-Lipoic acid (ALA) (purity ≥ 99%) specifically, was ordered from Sigma Aldrich (24899963). Ethanol (99.5%) was used as a solvent for ALA and, therefore, as a control in the experiments.

### 4.2. Cell Lines

Cells used for all experiments were SH-SY5Y-MOCK and SH-SY5Y-APP_695_. The neuroblastoma cell line SH-SY5Y was transfected with the amyloid-precursor protein 695; this is a well-established model for early AD [30,71]. SH-SY5Y-MOCK cells transfected with the empty vector pCEP4 served as a control group. The cells were kindly donated by A. Eckert (Basel, Switzerland). SH-SY5Y cells were cultivated in a humidified incubator at 37 °C containing 5% CO_2_. Cells were cultivated in 250 mL Greiner flasks with Dulbecco’s Modified Eagle Medium (DMEM) (Gibco Thermo Scientific, Grand Island, NY, USA) supplemented with 10% (*v*/*v*) heat-inactivated fetal bovine serum (FBS), 5 mL vitamins, 5 mL not essential amino acids (NEAA), 1 mM sodium pyruvate, 0.3 mg/mL hygromycin, 60 units/mL penicillin, 60 μg/mL streptomycin, and 4500 mg/l D-glucose. To prevent overgrowth, cells were transferred to new culture flasks once their confluency reached 70–80%. Two days before incubation, cells were harvested from Greiner flasks and seeded either into 24-well plates (MMP, 2 × 10^5^ cells/well) or 96-well plates (ATP, 2 × 10^4^ cells/well) in reduced DMEM (2% FBS, other components identical to growth medium). After 48 h, cells were incubated with ALA in various concentrations and ethanol as a control for 24 h. To investigate the effect of ALA on cell damage, a subset of cells were additionally exposed to 25 µM rotenone 1 h after ALA or EtOH exposure.

### 4.3. Measurement of ATP Concentrations

ATP concentrations were determined using the ATPlite Luminescence Assay System (Perkin Elmer, Rodgau-Jügesheim, Germany). This assay utilizes the light emission that occurs when ATP is combined with luciferin. The emitted light was evaluated with a ClarioStar plate reader (BMG Labtech, Ortenberg, Germany). Plates were removed from the incubator to cool down to room temperature for 15 min. Subsequently, lysis buffer was added and after 5 min cells were incubated with monitoring reagent for an additional 40 min in the dark. The results were normalized to the cell count.

### 4.4. Measurement of Mitochondrial Membrane Potential (MMP)

MMP levels were determined using the fluorescent dye rhodamine 123 (R123). After 24 h of incubation with the test substances, both cell lines were incubated for 15 min at 37 °C and 5% CO_2_ with 0.4 µM R123. The cells were centrifuged at 240 g for 5 min, washed, and resuspended in Hank’s Balanced Salt Solution (HBSS) (supplemented with Mg^2+^, CA^2+^, and HEPES; pH 7.4; 37 °C). The fluorescence of R123 was determined using a ClarioStar plate reader (BMG Labtech, Ortenberg, Germany) with an excitation wavelength of 490 nm and an emission wavelength of 535 nm.

### 4.5. High-Resolution Respirometry

To evaluate the oxygen consumption of the ETC an Oxygraph-2k respirometer (Oroboros Instruments, Innsbruck, Austria) and the software DatLab v. 4.3.2.7 was used (Oroboros, Innsbruck, Austria). The protocol used to test mitochondrial respiration was developed by Greiner [72]. For this, cells were harvested from 75 mL Greiner flasks following a 24 h long incubation with ALA or control after reaching a confluency of 70–80%. Cells were washed with PBS (1×) and diluted to 10^6^ cells/mL in a mitochondrial respiration medium (MIR05) [73]. At different stages of the experiment, the addition of various inhibitors, substrates, and uncouplers were used for direct investigation of individual ETC complexes. Endogenous respiration was determined by adding 2 mL cell suspension into the chambers. To permeabilize the plasma membrane, 1 µg digitonin per 10^6^ cells was added, which leaves the mitochondrial membranes intact. To compensate for proton leak through the membrane, substrates glutamate [10 mM] and malate [2 mM] were added and the leak respiration was measured (CI_(L)_). The addition of ADP [2 mM] was used to determine coupled complex I respiration CI_(P)_. CI and CII respiration in the coupled state (CI&CII_(P)_) was measured after the addition of succinate [10 mM]. Subsequent titration of carbonyl cyanide p-trifluoromethoxy phenylhydrazone (FCCP), of up to 2.5 µM, led to uncoupling of the ETC, and the maximal uncoupled activity of CI&CII_(u)_ was measured. After the addition of 0.5 µM rotenone uncoupled CII_(u)_ respiration was monitored. By the addition of oligomycin [2 µg/mL], the leak respiration of CII_(L)_ was determined. To exclude the oxygen consumption from enzymes that are not part of the oxidative phosphorylation, antimycin A was added and subtracted from all readings obtained in the experiment. By adding the electron donor N,N,N′,N′-tetramethyl-p-phenylenediamine dihydrochloride (TMPD) [0.5 mM], an artificial substrate for CIV, and ascorbate, which serves to keep TMPD in a reduced state, the maximal uncoupled respiration of CIV_(u)_ was measured. At the end of the experiment, NaN_3_ was added to inhibit CIV. The remaining oxygen consumption was due to the autooxidation of TMPD and was additionally subtracted from the raw CIV readings.

### 4.6. Citrate Synthase Activity

To assess the citrate synthase activity, cell samples from the respiration measurements were previously frozen in liquid nitrogen and stored at −80 °C. The following steps were taken to determine the CS activity. (1) Cells were mixed with a reaction medium containing 5,5′-dithio-bis-(2-nitrobezoic acid) (DTNB) [0.1 mM], acetyl coenzyme A [0.31 mM], EDTA [50 µM], oxalacetate [0.5 mM], triethanolamine hydrochloride [5 mM], and Tris HCl [0.1 M]. (2) The reaction medium was warmed to 30 °C for 5 min. (3) Cells were mixed with this medium. On the basis of conversion from DTNB into TNB, the citrate synthase activity was spectrophotometrically determined at a wavelength of 412 nm [74].

### 4.7. Protein Content

To determine protein content, Pierce BCA Protein Assay Kit (Thermo Fisher Scientific) was used according to the manufacturer’s instructions. The measurements were carried out on a CLARIOstar plate reader (BMG Labtech). Each sample was measured in triplicates.

### 4.8. ROS Measurement

Cellular ROS production was determined using a DCFDA/H2DCFDA Cellular ROS Assay Kit (ab113851; Abcam, Cambridge, UK). Cells were incubated with ALA for 24 h. Afterwards, the manufactures instructions were followed.

### 4.9. Statistics

Unless stated otherwise, data are presented as mean ± standard error of the mean (SEM). Statistical analyses were performed using either one-way analysis of variance (ANOVA) with Tukey’s multiple comparison post-hoc test or student’s *t*-test (Prism 8.0 GraphPad Software, San Diego, CA, USA). Statistical significance was defined for p values as * *p* < 0.05, ** *p* < 0.01, *** *p* < 0.001, and **** *p* < 0.0001.

## 5. Conclusions

In neuronal cells serving as a model for early AD and its controls, low ALA concentrations increased the activity of respiratory chain complexes and consequently ATP levels. Thus, ALA can compensate for mitochondrial dysfunction in cells that is not disease related. The increased activity of the mitochondrial respiratory complexes led to an increased formation of ROS, which can be intercepted by ALA and, thus, protect the mitochondria from oxidative stress. To better understand the observed positive results of ALA, the morphology and structure of mitochondria should be examined. Moreover, mitochondrial-associated factors should be further explored. In the future, in vivo validation of the ALA results should be performed.

## Data Availability

The data presented in this study are available on request from the corresponding author.
